# Outcome of Monochorionic Pregnancies after Selective Feticide with Bipolar Cord Coagulation: A German Single Center Experience

**DOI:** 10.3390/jcm11061516

**Published:** 2022-03-10

**Authors:** Eva Christin Weber, Brigitte Strizek, Florian Recker, Annegret Geipel, Ulrich Gembruch, Christoph Berg, Ingo Gottschalk

**Affiliations:** 1 Division of Prenatal Medicine, Gynecological Ultrasound and Fetal Surgery, Department of Obstetrics and Gynecology, University of Cologne, 50937 Cologne, Germany; prof.berg@icloud.com (C.B.); ingo.gottschalk@uk-koeln.de (I.G.); 2 Department of Obstetrics and Prenatal Medicine, University of Bonn, 53127 Bonn, Germany; brigitte.strizek@ukbonn.de (B.S.); florian.recker@ukbonn.de (F.R.); annegret.geipel@ukbonn.de (A.G.); ulrich.gembruch@ukbonn.de (U.G.)

**Keywords:** selective feticide, bipolar cord coagulation, monochorionic twin pregnancy, selective growth restriction, discordant anomaly

## Abstract

Objectives: To review the outcome of complicated monochorionic pregnancies after fetoscopic selective feticide with bipolar cord coagulation in an experienced German center. Methods: All cases that underwent selective feticide using fetoscopic bipolar umbilical cord occlusion (and simultaneous dissection in monoamniotics) at the University of Bonn in the past 10 years were retrospectively analyzed for antenatal and neonatal course and outcome. An adverse outcome was defined as either intrauterine death (IUD), neonatal death (NND), preterm prelabour rupture of membranes (PPROM), or preterm delivery (PTD) before 32.0 weeks of gestation. Results: We diagnosed 56 monochorionic pregnancies, including 43 diamniotic and 8 monoamniotic twins, as well as 5 triplets, complicated by discordant fetal anomalies (*n* = 10), selective intrauterine growth restriction (*n* = 29), twin-to-twin transfusion syndrome (*n* = 13), twin reversed arterial perfusion sequence (*n* = 3), or severe early twin anemia polycythemia sequence (*n* = 1), that underwent fetoscopic selective feticide in the 10 years study period. Selective feticide was performed by bipolar cord coagulation at a median gestational age of 21.2 weeks. PPROM occurred in 11 cases, 7 (12.5%) before 32.0 weeks and 4 (7.1%) between 34.0 and 36.0 weeks, respectively. There were five (8.9%) co-twins IUDs at a median of 2 weeks after the intervention. We observed 12 (21.4%) PTDs before 32.0 weeks of gestation and 2 (3.6%) NNDs. Mean gestational age at delivery was 37.1 weeks, with an overall survival of the co-twin of 87.5%. Conclusion: In experienced hands, fetoscopic selective feticide is an effective treatment in complicated monochorionic pregnancies. By sacrificing a sick fetus that jeopardizes the entire pregnancy, a higher survival rate of the co-twin can be achieved.

## 1. Introduction

Monochorionic (MC) twin pregnancies can be complicated by several problems, such as discordant congenital anomalies (DA), selective intrauterine growth restriction (sIUGR), twin-to-twin transfusion syndrome (TTTS), twin reversed arterial perfusion (TRAP) sequence or twin anemia-polycythemia sequence (TAPS) [1,2]. These conditions may jeopardize the entire pregnancy due to the presence of placental vascular anastomoses. In the case of fetal demise of one twin, the co-twin is at 60% risk for adverse outcomes, including co-twin death, brain damage and preterm delivery [3,4]. Inter-twin blood exchange via the placental anastomoses also prohibits the use of intravascular potassium chloride for feticide, as any substance injected into one fetus’ circulation may transit to the healthy co-twin. Furthermore, the anastomoses may cause the acute hemorrhage of the co-twin into the dying fetus [5]. Therefore, numerous techniques for selective feticide are described to ensure a complete disconnection of the two circulations [6,7,8,9,10,11,12]. The best survival rates for the normally developed twin are reported for cord occlusion. This can be achieved either by laser coagulation, radiofrequency ablation, or bipolar coagulation. The latter technique is the most effective at a higher gestational age when the umbilical cord is thicker. In Germany, only a few centers offer cord coagulation in twins. Therefore, the purpose of this study was to compare our results with other international centers.

## 2. Material and Methods

All cases of complicated MC pregnancies that underwent selective feticide with bipolar coagulation between January 2011 and January 2021 at the University Hospital of Bonn were retrospectively analyzed. In dichorionic triplet pregnancies that underwent selective feticide of the complicated MC pair, we analyzed only the MC pair. MC triplets and higher-order pregnancies were excluded. In each case, a detailed scan was performed, including biometric measurements, Doppler velocity flow profiles in the umbilical artery (UA), middle cerebral artery (MCA) and ductus venosus (DV), and a meticulous scan for structural malformations. All patients were counselled about the condition and the different options of management. Selective feticide was offered to the following patients:

- Severe discordant anomaly (DA): when the anomaly was a threat to the entire pregnancy (e.g., severe polyhydramnios) or parental decision for feticide of the abnormal fetus.
- sIUGR: If worsening of the Doppler measurements was observed with absent or reverse end-diastolic flow (AEDF or REDF) in either the UA or the DV prior to viability.
- Severe TTTS when laser therapy seemed not feasible: either the acceptor was suffering cardiac failure with abnormal flow in the DV, atrioventricular regurgitation, brain damage or hydrops fetalis, or the donor was presented with the reversed end-diastolic flow in the UA.
- TRAP: any case of TRAP as the risk of the fetal demise of the pump twin is high even in the absence of ultrasound finding, suggestive for high cardiac output failure
- TAPS: severe, early TAPS, inaccessible by fetoscopy, or laser therapy.

All ultrasound examinations prior to the intervention as well as for the follow-up were performed using 5-MHz or 7.5-MHz probes on high-end ultrasound systems, either Voluson E8 and E10 (GE Healthcare, Solingen. Germany), IU22 and Epiq 5 and 7 (Philips, Hamburg, Germany), or Aplio 500 and 600 (Canon/Toshiba Medical Systems, Neuss, Germany).

**Figure 1:**
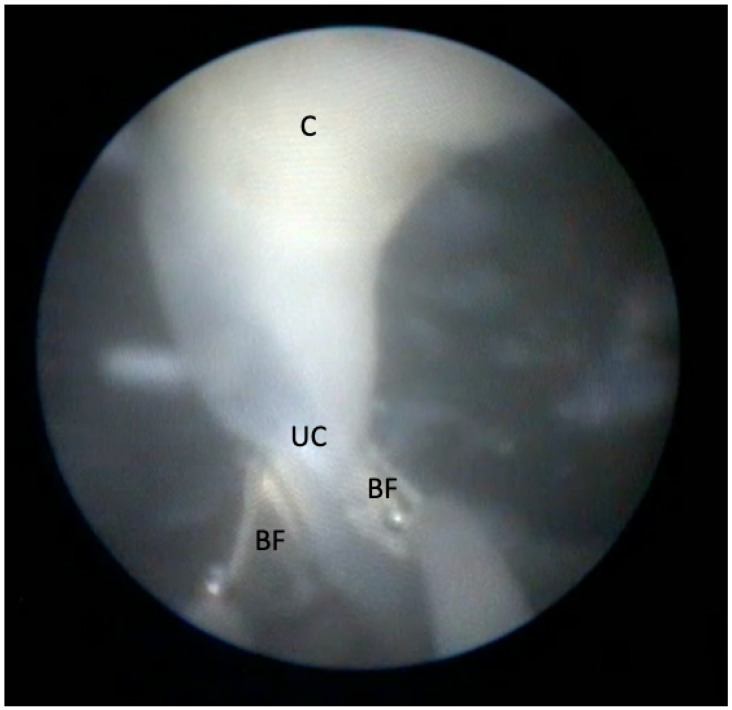
Occlusion of the umbilical cord (UC) with bipolar forceps (BF) at multiple levels. The level of the preceding coagulation (C) is depicted at 12 o’clock.

**Figure 2:**
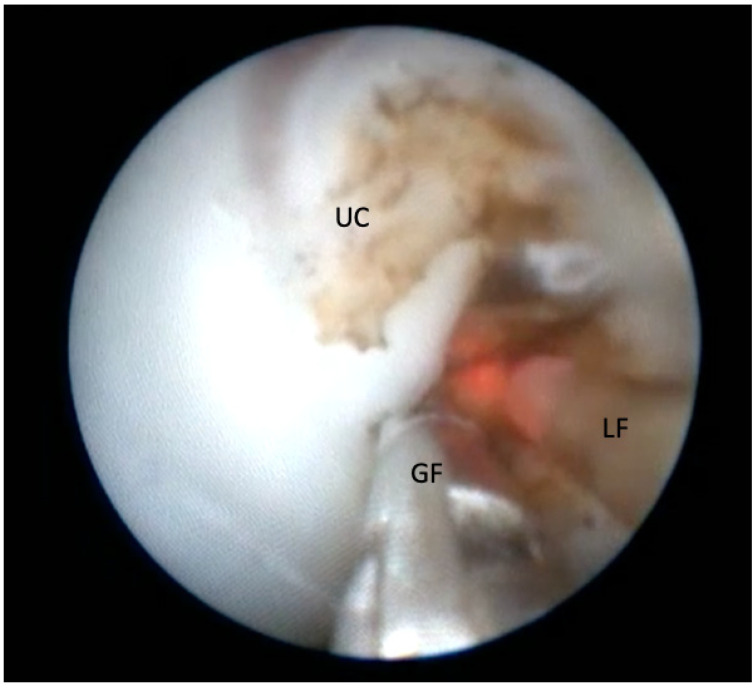
Dissection of the coagulated umbilical cord (UC) with a 3 Charr. grasping forceps (GF) and a bare laser fiber (LF).

In MC diamniotic pregnancies, feticide was performed by cord occlusion using bipolar optical coagulation forceps (11540 FG, Storz, Tuttlingen, Germany) introduced through an 11 Fr introducer as previously described (Figure 1) [13]. We avoided septostomy in all procedures. When oligohydramnios was present in the target sac, we used warmed saline 0.9% to fill the sac and allow vision throughout the procedure. When the amniotic fluid was blood-stained or dense, we exchanged the fluid until the vision was acceptable. In MC monoamniotic pregnancies, we performed cord occlusion and dissection. Cord occlusion was achieved as described above, and the dissection was performed using a 0.7 mm laser fibre in contact mode on the free umbilical cord until 2017. Thereafter, following the report of Greimel et al. 2018, we used a 3 Charr. grasping forceps (11510 C, Storz, Tuttlingen, Germany) to allow controlled manipulation of the umbilical cord during the procedure resulting in shorter intervention time (Figure 2) [14].

All interventions were performed by two surgeons (C.B. and B.S.) with extensive experience in intrauterine interventions. Daily ultrasound scans were performed until up to 96 h after the intervention to assess the Doppler and cardiac function of the pump twin. The majority of patients were discharged 2 days after the intervention. Regular follow-up visits were scheduled at least every 4 weeks.

The following obstetric data were recorded: indication for selective feticide, technique used, gestational age at presentation and intervention, pregnancy course, gestational age at delivery and mode of delivery, birthweight, neonatal mortality, and morbidity. An adverse outcome was defined as intrauterine death (IUD), preterm premature rupture of membranes (PPROM), preterm delivery (PTD) before 32.0 weeks of gestation, or neonatal death (NND). Miscarriages before 23.0 weeks of gestation were considered as IUD.

Statistical analysis was performed using descriptive statistics for baseline characteristics and the Fisher exact test for differences between categorical variables. All values are given as a median ± (range 25. quartile (Q1) to 75 quartile (Q3)) unless otherwise indicated. A *p*-value < 0.05 was considered significant. The statistical software used was IBM SPSS Statistics for iOS, version 27.0 Armonk, NY: IBM Corp.

Ethical approval was granted by the institutional ethics committee of the University of Bonn (Lfd. Nr. 5525/20).

## 3. Results

**Figure 3:**
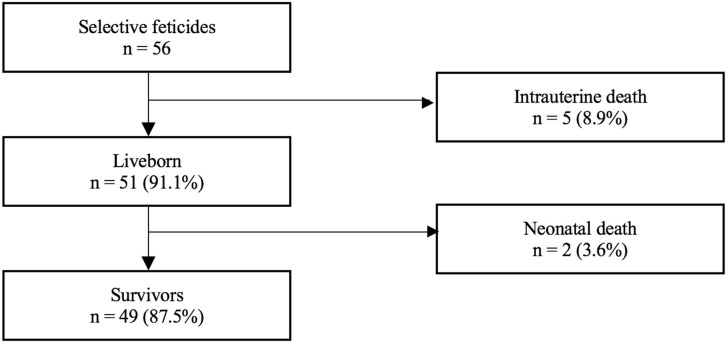
Mortality rate of 56 monochorionic cases that underwent selective feticide.

In the study period, 56 MC pregnancies underwent selective feticide. Eight of them were monoamniotic pregnancies, including two cases that were part of a previous study [15]. Five cases were triplets complicated by an MC pair. Figure 3 shows the overall mortality rate of our cohort treated with selective feticide.

Median gestational age at first presentation was 20.2 (interquartile range (IQR), Q1–Q3: 17.5 to 22.3) weeks and median gestational age at intervention was 21.2 (IQR, Q1–Q3: 19.5 to 23.1) weeks of gestation. Table 1 shows the general information on the indications for selective feticide in our cohort.

Of the 56 cases, intrauterine death (IUD) of the co-twin or miscarriage occurred in 5 (8.9%) cases at a median of 2.0 (IQR, Q1–Q3: 0.0 to 5.2) weeks after the intervention. This includes two co-twins that died within the first 24 h after intervention. The remaining 51 (90.1%) co-twins were born alive at a median gestational age of 37.1 (IQR, Q1–Q3: 32.0 to 38.3) weeks of gestation. Among them, 12 deliveries occurred prior to 32.0 weeks of gestation, including 2 deliveries at 23.0 and 24.3 weeks, respectively. Both of the latter co-twins died within the neonatal period due to severe prematurity. PPROM occurred in 11 pregnancies, including 7 cases before 34 weeks. The overall survival rate was 87.5%.

Table 2 shows the characteristics and outcomes of all 56 pregnancies that underwent selective feticide. There were no significant statistical differences in the characteristics of the five groups.

Of the 56 cases that underwent selective feticide, there were 8 monoamniotic twins and 5 dichorionic triamniotic triplets.

The indication for the intervention in the eight monoamniotic couples was fetal anomaly in three cases, TTTS in two cases and TRAP in three cases. There were two IUD at 21.2 and 20.5 weeks of gestation and three PTD at 26.0, 26.4, and 33.3 weeks of gestation. The remaining three cases ended in term delivery at 38.0, 38.5, and 39.0 weeks of gestation.

The five dichorionic triamniotic triplet pregnancies underwent selective feticide of the MC pair because of fetal anomaly, TTTS and sIUGR in one, one, and three cases, respectively. There was no IUD of either the MC co-twin or the singleton. We observed four PTD at 25.2 weeks, 29.2 weeks, 29.6 weeks, and 33.4 weeks. Three of those PTD occurred after PPROM. Of the triplet pregnancies, only one ended in term delivery at 38.5 weeks of gestation.

If monoamniotic twins and dichorionic triamniotic triplet pregnancies are excluded, the survival rate in our cohort is 88.4% (38/43), the rate of PPROM before 34.0 weeks of gestation 7.0% (3/43), and the rate of PTD before 32.0 weeks of gestation 16.3% (7/43).

## 4. Discussion

Our study demonstrates an 87.5% survival rate of the co-twin after selective feticide with bipolar cord coagulation in a cohort with various indications for this intervention. This compares well to other international centers specialized in fetal surgery.

In multiple pregnancies, the likelihood of finding any anomaly in at least one fetus is multiplied. MC pregnancies carry an additional risk for fetal demise due to their inter-twin placental vascular anastomoses; therefore, they jeopardizing the entire pregnancy. In the hands of specialists, selective feticide is an effective treatment for some of the complications related to MC pregnancies. In our cohort, we evaluated pregnancy course and perinatal outcome after selective feticide in MC pregnancies, performed by bipolar cord coagulation (*n* = 56) in a large German center. The overall survival rate in our cohort was 87.5% which is comparable with other high volume centers for fetal surgery in the USA, Israel, Switzerland, and Italy [16]. Two leading European tertiary fetal medicine centers in Belgium and Spain described survival rates of 83% after selective feticide [17].

A systematic review of 345 complicated MC pregnancies treated with selective feticide reported an overall survival of 79% [18]. In that meta-analysis, survival rates were highest in those pregnancies that underwent radiofrequency ablation (86%) and bipolar cord coagulation (82%), compared with laser cord coagulation (72%) and cord ligation (70%). Their analyses also showed higher survival rates if the intervention was performed after 18 weeks of gestation in comparison with earlier interventions (89% vs. 69%, *p* = 0.02). We had the same experience in our center in recent years and treated all patients in this cohort by bipolar coagulation after 18 weeks of gestation (with the exception of one TRAP pregnancy at 16.1 weeks of gestation). The low mortality of 3.6% in our report is comparable with the 7% mortality rate reported by Rossi et al. [18]. It may even be lower, as this review contained 26 pregnancies that underwent early selective feticide prior to 18 weeks. However, over the past decade, we preponed many of our interventions in monochorionic TRAP pregnancies into the first trimester using an intrafetal laser, with promising results [13]. Therefore, all TRAP pregnancies included in this cohort were diagnosed at a later gestational age, when an intrafetal laser is not suitable anymore. The low survival rate in TRAP pregnancies of 33.3% is most likely due to the small sample size, as we only included three cases of TRAP in our cohort. Furthermore, all of these cases were monoamniotic, carrying higher risks for complications. The expected survival rate of the pump twin after ablation of the TRAP in the literature is 80% [19] and therefore comparable to the overall survival in this study.

There were two neonatal deaths in our cohort. Both occurred in pregnancies with sIUGR due to extremely premature delivery at 23.0 and 24.3 weeks of gestation. In those cases, delivery was prompted by amnion infection and premature contractions. As described in previous studies [20], PPROM and preterm delivery are the major causes for adverse outcomes. Four of the eleven PPROMs in our cohort occurred after 34 weeks of pregnancy and therefore did not result in severe prematurity. However, almost half of our treated pregnancies (44.6%) resulted in premature deliveries, including 21.4% early premature deliveries before 32.0 weeks, resulting in increased neonatal morbidity.

It has to be considered that 13 (8 monoamniotic twins and 5 dichorionic triamniotic triplets) of our 56 cases were at an even higher risk, not only by the intervention but also due to the complicated nature of the pregnancy itself. Almost half of the IUD in our cohort (2/5) occurred in the monoamniotic co-twin, and almost one-third of the PTD in our cohort (7/11) were observed in the monoamniotic co-twin or the triplets. Excluding that subgroup from analysis renders the outcome of our cohort distinctly better.

Despite being one of the leading national centers, the number of treated pregnancies in our cohort is small due to the rarity of the disease. In addition, the underlying reason and the indication for selective feticide differ largely within our cohort, rendering the numbers of subgroups even smaller. The retrospective study design with its common weaknesses and the short follow-up are further limitations of our study. Furthermore, we cannot offer any information on the co-twin’s development past the neonatal period.

In conclusion, our study shows that our results are comparable to other international specialized centers, treating complicated MC pregnancies with selective feticide. Tailored treatment should be in the hands of experts in order to optimize outcomes. The risk of PPROM and PTD are relevant, but considering the risks carried for the healthy co-twin without intervention, it is feasible. The aforementioned risks are even higher in triplet or monoamniotic pregnancies. In complicated MC twins diagnosed after the first-trimester, bipolar cord coagulation for selective feticide seems to be safe and effective from 18 weeks onward.

## Figures and Tables

**Table 1:** Five groups according to indication for selective feticide.

Indication	All (*n*)	DA Twins (*n*)	MA Twins (*n*)	DC TA Triplets (*n*)
Discordant anomaly	10	6	3	1
Selective IUGR	29	26	0	3
Type I	4	4	0	0
Type II	16	15	0	1
Type III	9	7	0	2
TTTS	13	11	2	1
TTTS II°	2	2	1	1
TTTS III°	4	4	0	0
TTTS IV°	6	5	1	0
Recipients	8	6	1	1
Donors	5	4	1	0
TRAP sequence	3	0	3	0
TAPS	1	1	0	0

DA, diamniotic; DC, dichorionic; IUGR, intrauterine growth restriction; MA, monoamniotic; TA, triamniotic; TAPS, twin anemia polycythemia sequence; TRAP, twin reversed arterial perfusion; TTTS, twin-twin transfusion syndrome.

**Table 2:** Characteristics and outcomes of 56 cases after selective feticide, given in median (interquartile range (IQR), Q1–Q3).

Characteristic	All *n* = 56	Discordant Anomaly *n* = 10	sIUGR *n* = 29	TTTS *n* = 13	TRAP Sequence *n* = 3	TAPS *n* = 1
GA at presentation (weeks)	20.2 (17.5–22.3)	21.6 (16.7–25.3)	20.1 (17.3–22.4)	19.5 (18.9–21.7)	18.5 (14.1–n.a.)	21.5
GA at intervention (weeks)	21.2 (19.5–23.1)	21.9 (19.1–29.4)	21.6 (20.6–23.3)	20.0 (19.2–22.7)	19.0 (16.1–n.a.)	21.5
IUD (weeks)	21.1 (20.1–21.7)	-	21.6 (21.1–21.6)	19.6	20.9 (20.5–n.a.)	-
Intervention until IUD (weeks)	1.4 (0.0–4.4)	-	1.8 (0.0–n.a.)	1.4	2.6 (0.0–n.a.)	-
PPROM (weeks)	32.5 (25.6–35.5)	36.2	32.0 (29.1–33.9)	30.0 (23.7–35.9)	-	-
23–34 weeks (*n*)	7	0	5	2		
34–36 weeks (*n*)	4	1	1	2		
Intervention until PPROM (weeks)	10.0 (3.5–15.3)	16.6	9.6 (6.6–10.4)	9.4 (2.8–16.1)	-	-
GA at delivery (weeks)	37.1 (32.0–38.3)	36.1 (32.6–37.6)	37.6 (31.1–38.5)	36.3 (34.6–38.0)	39.0	38.6
Intervention until delivery (weeks)	14.1 (7.6–17.4)	7.5 (4.3–17.5)	13.0 (8.5–17.6)	15.4 (12.3–17.1)	20.0	17.1
PTD (*n* {%})	25 (44.6%)	6 (60.0%)	12 (41.4%)	7 (53.8%)	-	-
≤28 weeks (*n* {%})	6 (10.7%)	1 (10.0%)	4 (13.8%)	1 (7.7%)		
28–31.6 weeks (*n* {%})	6 (10.7%)	1 (10.0%)	4 (13.8%)	1 (7.7%)		
32–36 weeks (*n* {%})	13 (23.2%)	4 (40.0%)	4 (13.8%)	5 (38.5%)		
Birth weight (g)	2500 (1840–3000)	2525 (1792–2940)	2500 (1450–3280)	2400 (1841–2633)	2402	2550
IUD	5 (8.9%)	0	2	1	2	0
NND	2 (3.6%)	0	2	0	0	0
PPROM	11 (19.6%)	1	6	4	0	0
Perinatal Survival	49 (87.5%)	10 (100%)	25 (86.2%)	12 (92.3%)	1 (33.3%)	1 (100%)

n.a., not applicable.

## Data Availability

The data presented in this study are available on request from the corresponding author.
